# Comparison of conventional and minimally invasive approaches of primary total knee arthroplasty: a network meta-analysis of randomized controlled trials

**DOI:** 10.1186/s12891-026-09712-1

**Published:** 2026-03-11

**Authors:** Min-Hwan Huh, Hong-Jin Kim, Young-Soo Shin

**Affiliations:** 1 College of Medicine, The Graduate School, Yonsei University, Seoul, Republic of Korea; 2 Department of Orthopaedic Surgery, Mokdong Hospital, Ewha Womans University School of Medicine, Seoul, Republic of Korea; 3 Suwon Korea Bone Orthopaedics, 2139, Seobu-ro, Jangan-gu, Suwon-si, Gyeonggi-do 16360 Republic Of Korea

**Keywords:** TKA, Minimally invasive approach, Conventional approach, Quadriceps-sparing approach

## Abstract

**Background:**

The purpose of this study was to evaluate the efficacy of conventional and minimally invasive approaches to total knee arthroplasty (TKA) by comparing the pain score, range of motion (ROM), and adverse effects. It was hypothesized that minimally invasive approaches would lead to superior outcomes.

**Methods:**

Randomized control trials comparing various approaches to TKA were identified in various literature databases from conception through December 31, 2022. A network meta-analysis (NMA) of relevant literature was performed to investigate which approaches showed better outcomes. In total, 42 studies were included in this study.

**Results:**

The main finding of this NMA was that the minimally invasive midvastus (MMV) approach led to better outcomes in terms of pain score (with a surface under the cumulative ranking curve [SUCRA] value of 80.0 and a mean rank of 2.2). Also, the minimally invasive quadriceps-sparing (MQS) approach led to better outcomes in terms of ROM (with a SUCRA value of 90.0 and a mean rank of 1.6). However, the subvastus (SV) and quadriceps-splitting (Qsplitt) approaches led to better outcomes in terms of reduction in adverse effects (SV: SUCRA value of 80.0 and a mean rank of 2.4; Qsplitt: SUCRA value of 70.0 and a mean rank of 3.1).

**Conclusion:**

According to this NMA, minimally invasive approaches of TKA led to better outcomes in terms of pain score and ROM, while conventional approaches of TKA led to better outcomes in terms of safety. Therefore, orthopedic surgeons should consider various factors when choosing the TKA approach.

**Trial registration:**

PROSPERO: CRD42024548966

## Background

Traditionally, the medial parapatellar (MP) approach is commonly used because it provides a wide surgical field of the knee joint for better prosthesis placement [1, 2]. However, the main disadvantages of this approach are violation of the extensor mechanism and injury to the vascularity of the patella [3]. Both events may lead to poorer functional outcomes, anterior knee pain, patellar maltracking, and fractures [4, 5]. To avoid these problems, the midvastus (MV) and subvastus (SV) approaches were developed to limit injury of the quadriceps tendon and avoid eversion of the patella [6, 7]. Furthermore, many minimally invasive approaches to reduce soft tissue damage and blood loss by limiting arthrotomy incision have been developed and studied [8–10]. The minimally invasive midvastus (MMV) or subvastus approaches and quadriceps-sparing (QS) approaches were developed to limit arthrotomy and better preserve the extensor mechanism [11–14]. However, minimally invasive approaches also carry adverse effects, such as longer surgical times, wound problems, or malalignment due to a limited visual field [15, 16]. Each approach has pros and cons, and controversy remains as to which approach is better [17, 18].

Although numerous studies have suggested which approaches are better between conventional and minimally invasive approaches, they often included small sample sizes, which can lead to reduced statistical power and conflicting results. Moreover, previous meta-analyses have only compared two or three approaches. A network meta-analysis (NMA) allows a unified, coherent analysis of data recorded in randomized controlled trials regarding the clinical effectiveness of all available treatment options based on evidence from direct and indirect comparisons of various treatments [19].

The purpose of the present study was to evaluate the efficacy of conventional and minimally invasive approaches by comparing the pain score, range of motion (ROM), and adverse effects. It was hypothesized that minimally invasive approaches would show superior outcomes.

## Methods

### Data and literature sources

This study followed the Preferred Reporting Items for Systematic Reviews and Meta-analyses (PRISMA) reporting guidelines for a network meta-analysis [20]. Although the current study involved human participants, ethical approval and informed consent from participants were not required because all data were acquired from previously published studies and analyzed anonymously without any potential harm to participants. MEDLINE, EMBASE, the Web of Science, SCOPUS, and the Cochrane Library were searched from conception to December 31, 2022, using logical keyword combinations and in-text words related to TKA. Articles written in English were collected. Following the initial electronic search, additional relevant articles and bibliographies were manually searched to identify eligible studies not captured by the initial database search.

### Study selection

For inclusion in this NMA, studies were required to (1) be randomized controlled trials including patients who underwent primary TKA without a history of previous knee surgery or major knee injury; (2) report on at least two of the approaches of MP, MV, SV, QS, MMP, MMV, minimally invasive subvastus (MSV), minimally invasive quadriceps-sparing (MQS), or quadriceps splitting (Qsplitt); (3) include a follow-up period ≥ 2 weeks after TKA; and (4) report surgical outcomes, including the postoperative pain score, degree of ROM, and numbers of patients who experienced adverse effects and severe adverse effects; (5) studies published in English. Studies were excluded if they were case reports or review articles or if they offered incomplete data.

### Data extraction

Two reviewers independently recorded data from each study using a predefined data-extraction form. Disagreement between the reviewers was resolved by consensus or by discussion with a third investigator. The recorded variables included information about the risk-of-bias assessment and outcome measures, as well as author name(s), publication year, patient demographics, sample size (total number of randomly assigned patients), comparators in the intervention groups, and intervention characteristics. We obtained the mean and standard deviation of pain scores and degrees of ROM from baseline and follow-up in each trial to calculate the mean change. If variances for net changes from baseline were not reported, they were calculated from standard errors, 95% confidence intervals (CIs), *P* values, or *t* statistics [21]. When no information was available to calculate the variances for paired differences, we imputed missing values using a correlation coefficient of 0.7 between baseline and follow-up scores because we assessed the sensitivity of our results relative to different correlation coefficients and confirmed the consistency of findings across sets of analyses.

### Methodologic quality assessment

Two reviewers independently assessed the methodologic quality of each study using a risk-of-bias table that included random sequence generation; allocation concealment; blinding of patients, surgeons, and outcome assessors; blinding of the outcome assessment; selective outcome reporting and other biases; and incomplete outcomes data as recommended by the Cochrane Bias Methods Group. The risk of bias (low, high, or unclear) was independently assessed by two investigators. In addition, the modified Jadad score was applied to evaluate randomizations, blindings, withdrawals and dropouts, inclusion and exclusion criteria, AEs, and statistical analyses. According to a previously published paper, studies scoring ≥ 4 of a total of 8 points were considered to be high quality [22]. All differences were resolved by two reviewers through discussion, and their decisions were subsequently reviewed by a third investigator. We used *k* values to evaluate the rater reliability for all items of the modified Jadad score.

### Outcome measures

Endpoint data, which included the mean changes from baseline in the pain score and ROM, were collected. If there was more than one time point reported in a window, we used the data closest to the longest follow-up duration in that window. The Knee Society Score (KSS) and the Hospital for Special Surgery Score (HSS), which are commonly used to measure pain intensity in TKA patients, were adopted for evaluation of pain scores [23, 24]. If the KSS or HSS was not reported, a visual analog scale was applied instead [25]. In this analysis, the majority of studies used the KSS to report pain outcomes, while a smaller number employed the HSS or VAS. To enable unified comparisons across studies, mean change values for pain-related outcomes were extracted and synthesized using ratio-based treatment effects in the network meta-analysis. The ROM measured in degrees was extracted. The number of patients who experienced adverse effects or severe adverse effects after TKA was collected. adverse effects of interest included deep vein thrombosis, nerve palsy, superficial hematoma, wound infection, and skin bulla, while severe adverse effects included fracture, bleeding (joint hematoma, hemarthrosis), reoperation (revision, manipulation, arthroscopic release), and joint infection. The definitions defined by the authors of the original article were used.

### Data analyses

We qualitatively synthesized included trials and then developed network diagrams to visualize the relative amount of available evidence on nine approaches [26]. In network diagrams, the size of each node and the thickness of each line connecting the nodes were proportional to the number of participants. For continuous endpoints, treatment effects were analyzed as ratios of mean changes with 95% CI using a random-effects model. The proportions of cases that developed adverse effects and severe adverse effects were calculated using a random-effects model as a binary endpoint, with weighted averages reported as odds ratios and associated 95% CIs. To test the NMA consistency assumption, we assessed the inconsistency factors based on the estimated difference between the effect size from direct comparisons within trials and the effect size from indirect comparisons within trials with one treatment in common. If the value approached 1, the two estimates were considered consistent with one another. We explored transitivity through inspection of the baseline data (both age and timepoint extracted) of the included trials. Furthermore, NMA is capable of ranking probability distributions of treatments generated from a simulation of 10,000 replications. The probability values are reported as the mean rank and surface under the cumulative ranking curve (SUCRA). The best treatment has a SUCRA value equal to 100%, whereas the worst treatment has a SUCRA equal to 0%. Publication bias was evaluated using funnel plots. In addition, sensitivity analyses were performed by excluding eligible trials to investigate the impact of the risk of bias on the result, with a high risk of bias identified according to the elements of randomization and blinding of the participants. We also measured the extent of heterogeneity with I² statistics with values interpreted as low (< 25%), moderate (25–50%), or high (> 50%) heterogeneity. All statistical analyses were performed using Stata version 14.2 (Stata Corp. LLC, College Station, TX, USA).

## Results

### Study characteristics

**Fig. 1:**
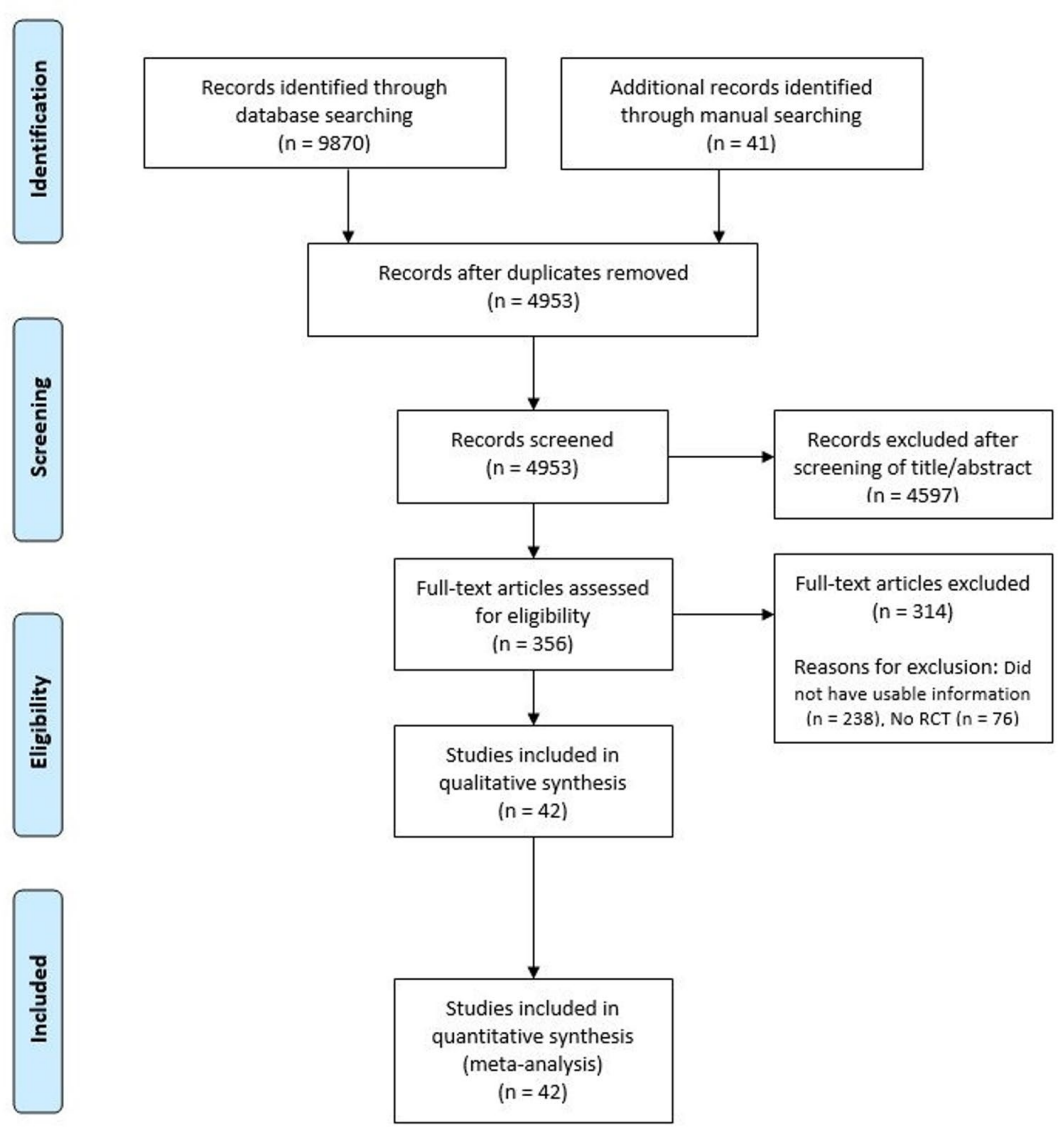
Flow diagram of study identification and selection

**Fig. 2:**
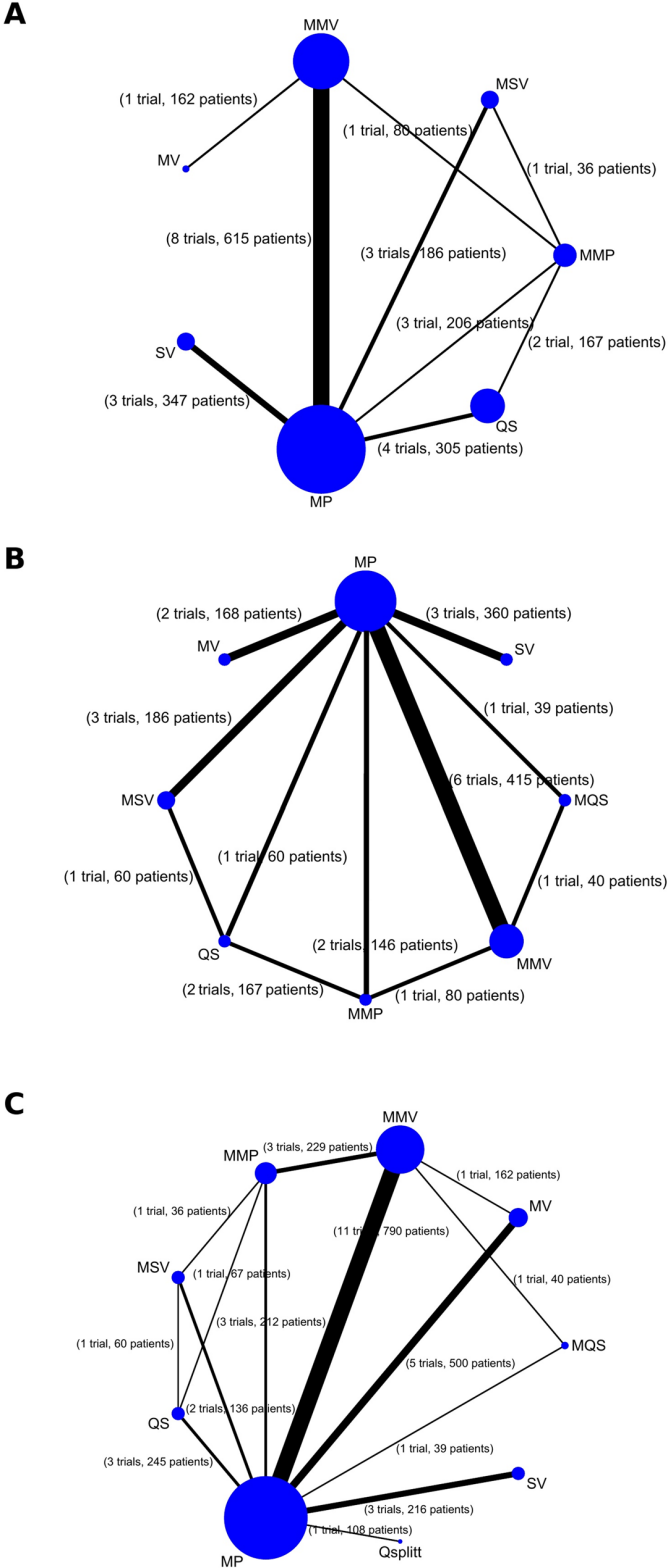
Structure of the network formed by interventions and their direct comparisons of pain score (**A**), range of motion (**B**), adverse effects and severe adverse effects (**C**). The lines between treatment nodes indicate the direct comparisons made within randomized trials. The numbers shown beside the lines represent numbers of trials/participants. *MP* Medial parapatellar, *MV* Midvastus, *SV* Subvastus, *QS* Quadriceps sparing, *MMP* Minimally invasive medial parapatellar, *MMV* Minimally invasive midvastus, *MSV* Minimally invasive subvastus, *MQS* Minimally invasive quadriceps-sparing, *Qsplitt* Quadriceps splitting

Details on study identification, inclusion, and exclusion are summarized in Fig. 1. A total of 42 studies was included in the NMA [3, 14, 27–66]. The network of interventions is shown in Fig. 2. In most studies, KSS pain scores were reported. The 35 studies randomly allocated their patients to one of nine approaches (MP, MV, SV, QS, MMP, MMV, MSV, MQS, or Qsplitt) and were published from 1997 to 2022. The length of follow-up ranged from two weeks to 70 months. The quality of the 42 studies included in this NMA is summarized in Table 1. Interrater reliabilities (*k* values) for all items of the modified Jadad score ranged from 0.81 to 0.93, suggesting substantial agreement between the two investigators. For evaluable analyses, funnel plots indicated a lack of publication bias among the included studies (symmetric for all). After we excluded trials with poor methodologic quality, no significant difference was identified compared to those of our primary analyses, indicating that the findings were robust to decisions made in the data-collection process. The I² values for heterogeneity were 11.3% for pain score, 18.5% for ROM, and 35.4% for adverse events, indicating low heterogeneity in the included studies.

**Table 1:** Characteristics and risk of bias and quality of included studies using a modification of the Jadad quality score assessment tool summary: review authors’ judgements about the risk of bias item for each included study

Study	Random sequence generation	Allocation concealment	Blinding of participant and personnel	Blinding of outcome assessment	Incomplete outcome	Existence of selective reporting	Existence of other bias	Modified Jadad scale	Mean age (years)	% women	Pain outcome extracted	Time point extracted (months)
Aglietti et al. 2006	-	+	-	-	-	?	?	7	70.5	65	VAS	3, 6
Bathis et al. 2005	+	+	-	-	-	?	?	6	69.6	76	VAS	0.5, 1.5
Bourke et al. 2012	-	-	-	-	-	?	?	6	67.9	59	KSS	18
Bridgman et al. 2009	-	-	-	-	-	?	?	7	70.5	48	KSS	1.5, 3, 12
Chiang et al. 2012	-	+	-	-	-	?	?	8	69.8	90	HSS	2, 24
Chin et al. 2007	+	+	-	-	-	?	?	5.5	65.4	85	KSS	6
Cho et al. 2014	+	+	+	+	-	?	?	5	66.3	95	KSS	1.5, 3, 6, 12
Dalury et al. 1999	+	+	-	-	-	?	?	5	70.0	66	VAS	1.5, 3
Dutton et al.2008	-	+	-	-	-	?	?	7	67.5	81	KSS	3, 6
Engh et al. 1997	+	+	+	+	-	?	?	3	69	65	NA	1.5
Geng et al. 2022	+	+	+	-	-	?	?	4	65	86	VAS	1
Guy et al. 2012	-	-	+	+	-	?	?	6	70.1	53	KSS	12
Heekin et al. 2014	+	+	-	-	-	?	?	4.5	65	35	KSS	3, 6, 12, 24
Hernandez et al. 2010	-	+	+	+	-	?	?	4	70.6	82	KSS	6
Huang et al. 2015	+	+	+	+	-	?	+	3	69.5	86	VAS	70
Jung et al. 2009	+	+	+	+	-	?	?	4	NA	92	NA	1.5, 6, 12, 36
Juosponis et al. 2009	-	-	+	+	-	?	?	4	71.7	85	KSS	3
Karachalios et al. 2008	-	+	-	-	-	?	?	7	71.0	66	KSS	3, 6, 9, 12, 24, 36
Karpman et al. 2009	-	-	-	-	-	?	?	7	73.3	59	VAS	6
Keating et al. 1999	+	+	-	-	-	?	?	4.5	70.2	60	NA	NA
Kelly et al. 2006	+	+	+	+	-	?	?	5	68.2	NA	HSS	6, 60
Kim et al. 2007	-	-	+	-	-	?	?	3	65.4	78	VAS	3, 12, 22
Kolisek et al. 2007	-	+	+	+	+	?	?	3	68.5	33	KSS	3
Lai et al. 2014	-	-	-	-	-	?	?	8	62.9	71	KSS	1.5, 3, 6, 9, 12, 24, 36
Lee et al. 2011	-	-	-	-	-	-	?	8	67	93	HSS	1, 3, 6
Lin et al. 2009	-	+	+	-	-	?	?	4	69.9	90	VAS	2
Lin et al. 2013	+	+	+	-	-	?	?	5	68.9	86	VAS	1, 24
Luring et al. 2008	-	+	+	+	-	?	?	6	69.3	NA	KSS	1.5, 6, 12
Nestor et al. 2010	+	+	+	+	-	?	?	4	66.7	67	VAS	1.5, 3
Pan et al.2010	-	-	-	-	-	?	?	7	62.8	71	KSS	1.5, 3, 6, 9, 12, 18
Pongcharoen et al. 2013	-	-	+	+	-	?	?	6	67	80	KSS	3, 6, 12, 24
Roysam et al.2001	-	-	+	-	+	?	?	3.5	70	47	NA	1, 3
Sastre et al. 2009	-	-	-	-	-	?	?	8	NA	NA	VAS	1, 3, 12
Seon et al. 2006	+	+	+	+	-	?	?	3	64.2	79	HSS	3, 6, 9, 12
Tashiro et al. 2007	+	+	+	+	-	?	?	2	78.0	90	KSS	3, 6, 12
Tasker et al. 2014	+	+	+	-	-	?	?	5.5	67.7	63	KSS	12, 24
van Hemert et al. 2011	+	+	-	-	-	?	?	5	70.6	68	KSS	1.5, 3
Varela-Egocheaga et al. 2010	-	+	+	+	-	?	?	5	69.3	73	KSS	1, 3, 12, 36
Wegrzyn et al. 2013	-	-	-	-	-	?	?	8	65.5	78	KSS	2
Yang et al. 2010	-	-	-	+	-	?	?	4.5	68.3	90	KSS	3, 6, 12
Zhang et al. 2013	-	+	+	+	-	?	?	5	63	69	KSS	1.5, 3, 6
Zora et al. 2020	+	+	+	-	-	?	?	5.5	64.1	93	VAS	1, 3

−, Low risk of bias; +, high risk of bias;?, unclear risk of bias. Modified Jadad score is applied to evaluate the quality of included studies, showing a score of ≥ 4 points are considered to be high quality. *VAS* Visual analog scale, *KSS* Knee society score, *HSS* Hospital for special surgery knee score, *NA* Not applicable

### Comparative effects on pain score

**Fig. 3:**
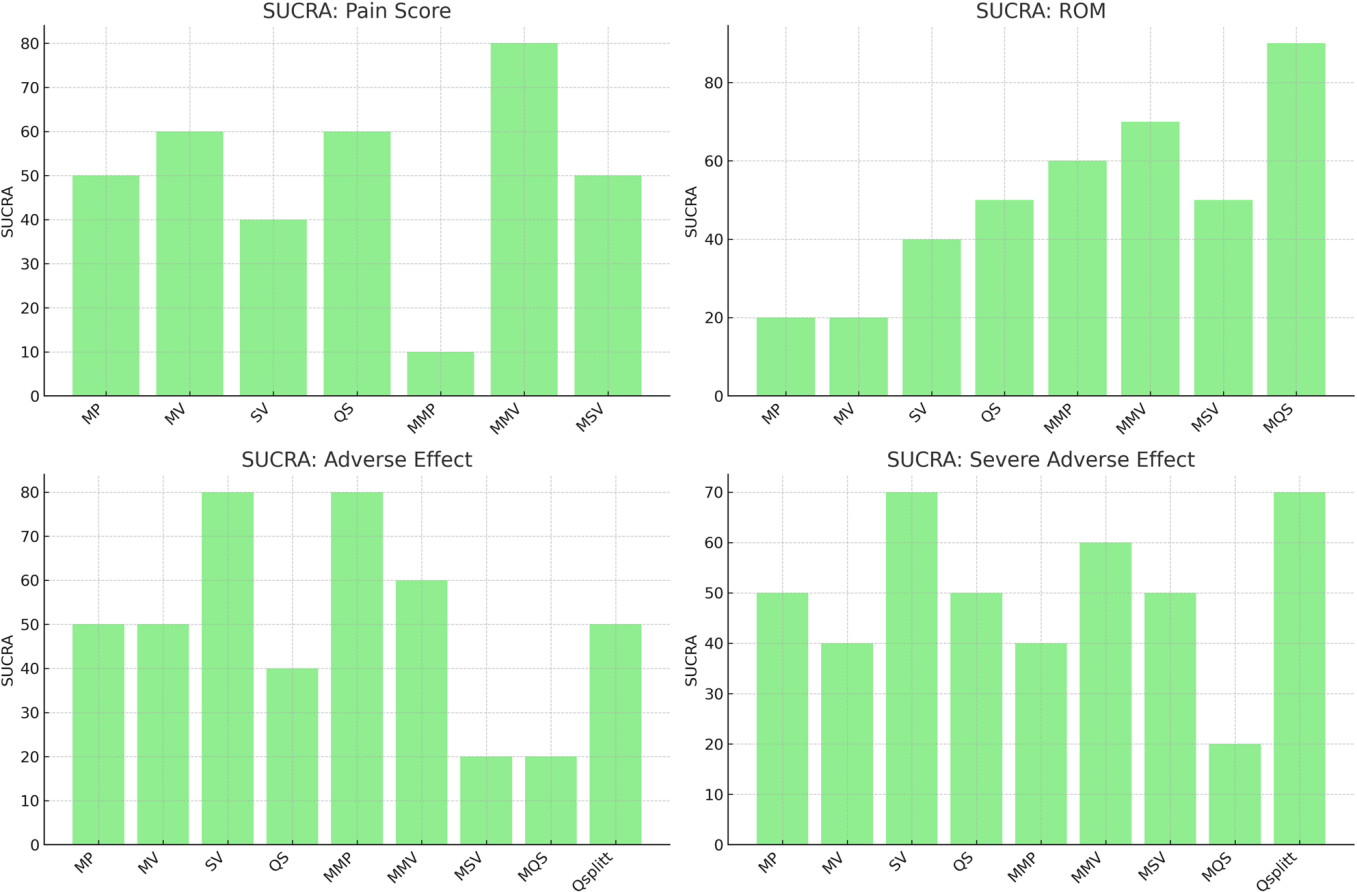
Graphical representation of SUCRA (Surface Under the Cumulative Ranking) values for each surgical approach based on network meta-analysis. Higher SUCRA values indicate a greater probability of being among the most effective or safest treatments for each outcome. SUCRA values represent the surface under the cumulative ranking probability curves derived from the network meta-analysis. *ROM* Range of motion, *MP* Medial parapatellar, *MV* Midvastus, *SV* Subvastus, *QS* Quadriceps sparing, *MMP* Minimally invasive medial parapatellar, *MMV* Minimally invasive midvastus, *MSV* Minimally invasive subvastus, *MQS* Minimally invasive quadriceps-sparing, *Qsplitt* Quadriceps splitting

**Fig. 4:**
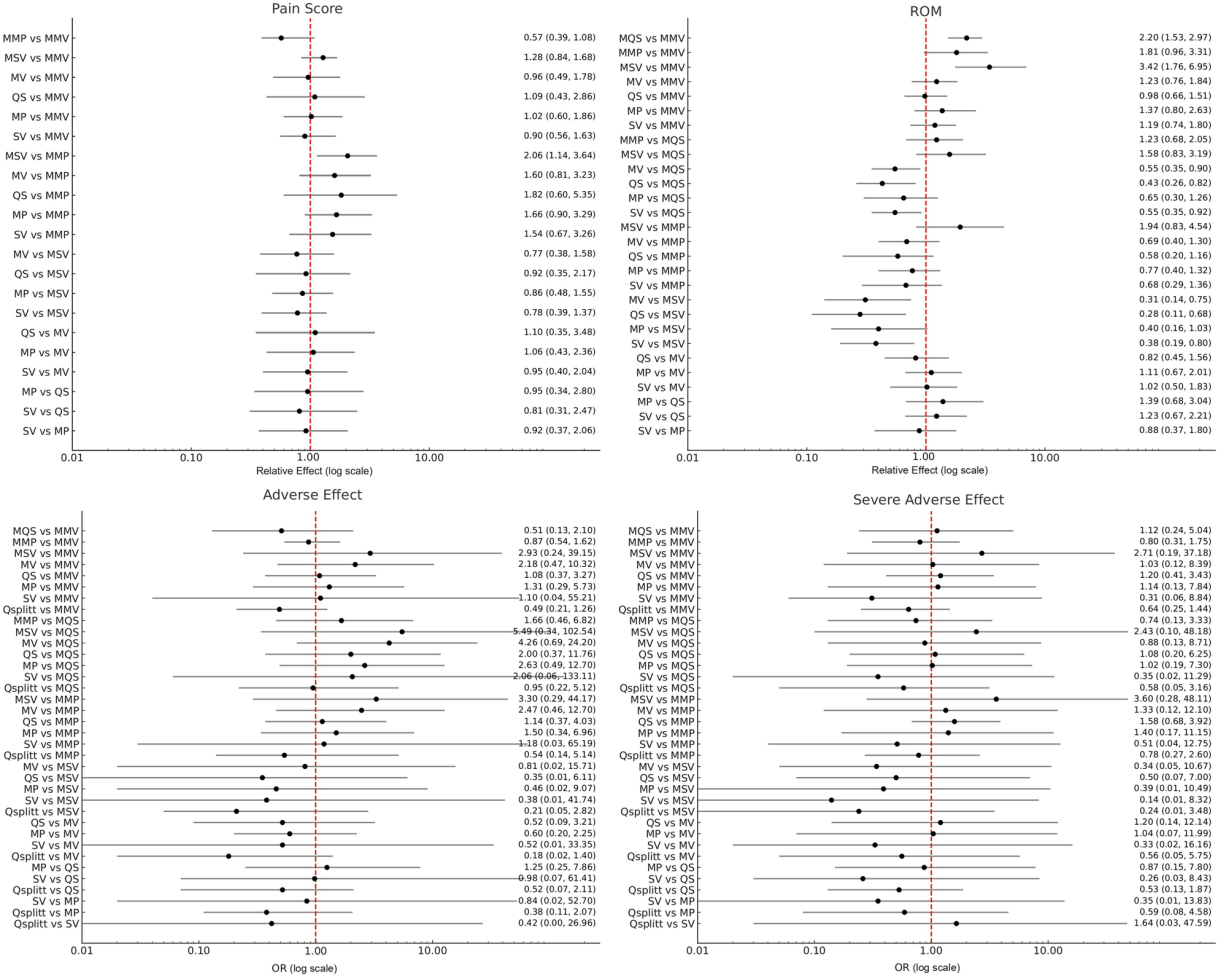
Interval plot presenting the relative treatment effects on a logarithmic scale and 95% confidence interval (CI) for pain scores, range of motion (ROM) outcomes, and odds ratios of adverse effects and severe adverse effects across different surgical approaches, based on network meta-analysis. The vertical dashed line at 1 indicates no difference between treatments. Values < 1 favor the comparator listed first, whereas values > 1 favor the comparator listed second. Because this study involves multiple treatment comparisons within a network meta-analysis framework, results are displayed as relative treatment effects rather than in a conventional pairwise forest plot format. *MP* Medial parapatellar, *MV* Midvastus, *SV* Subvastus, *QS* Quadriceps sparing, *MMP* Minimally invasive medial parapatellar, *MMV* Minimally invasive midvastus, *MSV* Minimally invasive subvastus, *MQS* Minimally invasive quadriceps-sparing, *Qsplitt* Quadriceps splitting, *OR* odds ratio

Of the 42 included studies, 26 with data suitable for pain score comparison after TKA were used for analysis. The SUCRA percentage showed that the MMV approach was most likely the best (with a SUCRA value of 80.0 and a mean rank of 2.2) and the MMP approach was most likely the worst (with a SUCRA value of 10.0 and a mean rank of 6.6) in terms of pain score. The SUCRA values and mean rank are summarized in Table 2. A graphical representation of the SUCRA values is provided in Fig. 3. Although most approaches were not significantly superior to one another, the MMP approach was significantly superior to the MSV approach in terms of pain relief. However, there was evidence of an inconsistency between direct and indirect estimates (*P* <.01). The comparative effectiveness results for pain score are shown in Table 3. The comparative effectiveness results for pain score are visualized using interval plots in Fig. 4.

**Table 2:** Network meta-analysis intervention ranking results for each of pain score, ROM, adverse effect, and severe adverse effect

Approaches	Pain score		ROM		Adverse effect		Severe adverse effect	
	SUCRA	Mean rank	SUCRA	Mean rank	SUCRA	Mean rank	SUCRA	Mean rank
MP	50	3.8	20	6.7	50	4.9	50	5.3
MV	60	3.4	20	6.6	50	5.1	40	6.2
SV	40	4.5	40	5.3	80	2.4	70	3.3
QS	60	3.6	50	4.3	40	5.6	50	5.4
MMP	10	6.6	60	3.7	80	2.8	40	5.6
MMV	80	2.2	70	3.4	60	4.3	60	4.2
MSV	50	4.0	50	4.6	20	7.3	50	5.2
MQS	NA	NA	90	1.6	20	7.1	20	7.4
Qsplitt	NA	NA	NA	NA	50	5.0	70	3.1

Surface under cumulative ranking curve (SUCRA) values (0–100) and mean ranks are presented, based on a simulation with 10,000 replications. Higher SUCRA s and lower mean ranks indicate better performing approach

*MP* Medial parapatellar, *MV* Midvastus, *SV* Subvastus, *QS* Quadriceps sparing, *MMP* Minimally invasive medial parapatellar, *MMV* Minimally invasive midvastus, *MSV* Minimally invasive subvastus, *MQS* Minimally invasive quadriceps-sparing, *Qsplitt* Quadriceps splitting, *NA* Not applicable, *ROM* Range of motion

**Table 3:**
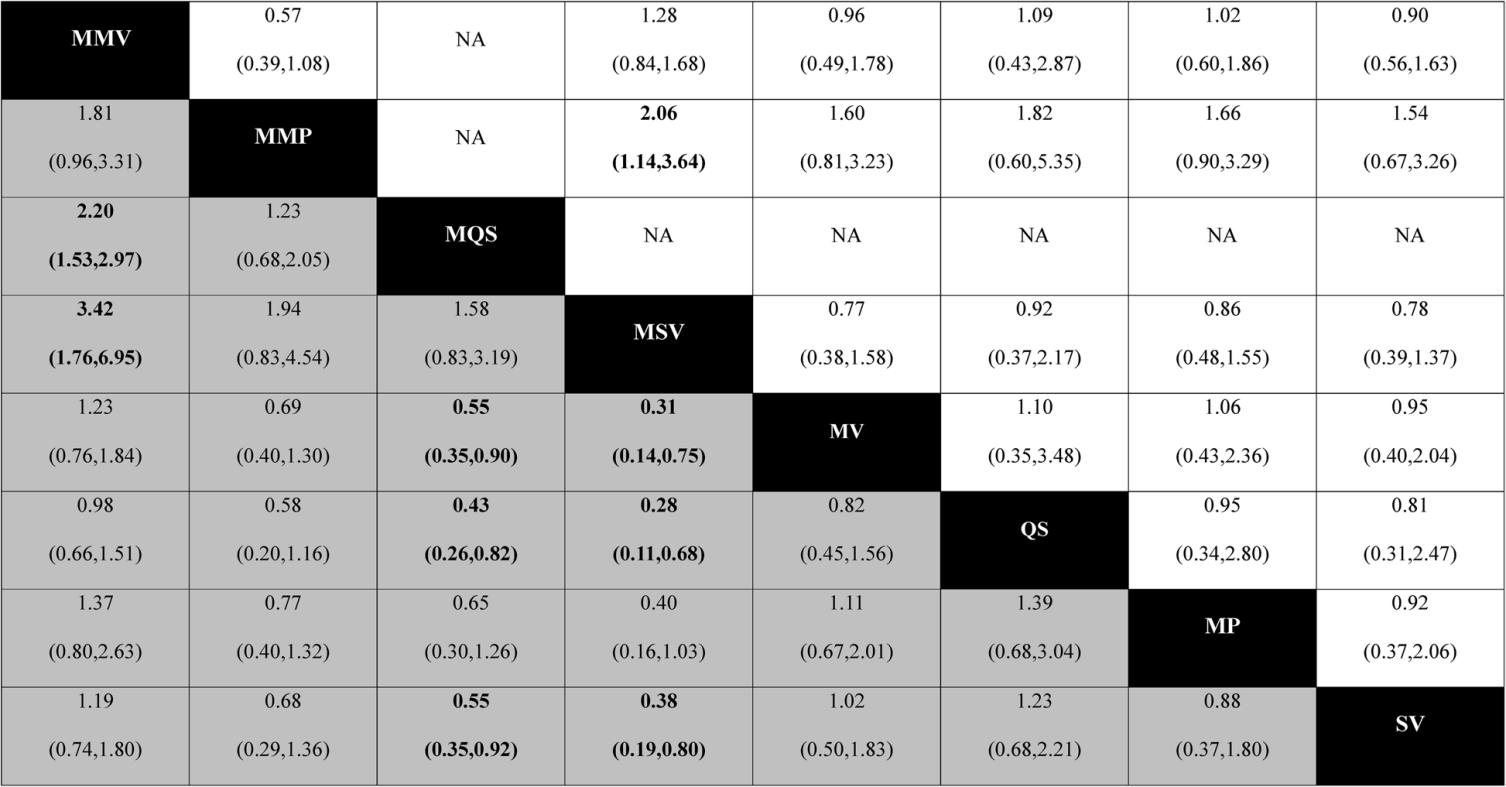
Network meta-analyses comparison between results of pain score (white) and range of motion (grey) at last follow-up time point from the baseline

Each cell shows the ratio of mean change with a 95% CI. Treatment effects were estimated on the logarithmic scale. A ratio > 1 favors the upper-left intervention, whereas a ratio < 1 favors the lower-right intervention

Significant results in bold text

*MP* Medial parapatellar, *MV* Midvastus, *QS* Quadriceps sparing, *SV* Subvastus, *MMP* Minimally invasive medial parapatellar, *MMV* Minimally invasive midvastus, *MQS* Minimally invasive quadriceps-sparing, *MSV* Minimally invasive subvastus, *NA* Not applicable, *CI* Confidence interval

### Comparative effects on ROM

Of the 42 included studies, 21 with data suitable for ROM comparison after TKA were used for analysis. The SUCRA percentage showed that the MQS approach was most likely the best (with a SUCRA value of 90.0 and a mean rank of 1.6) and the MP approach was most likely the worst (with a SUCRA value of 20.0 and a mean rank of 6.7) for ROM. The SUCRA values and mean rank are summarized in Table 2. A graphical representation of the SUCRA values is provided in Fig. 3. Although most approaches had similar effectiveness, the MMV approach was significantly superior to the MQS and MSV approaches. In addition, the MQS and MSV approaches were significantly inferior to the MV, QS, and SV approaches. However, there was inconsistency between direct and indirect estimates (*P* <.01). The comparative effectiveness results for ROM are shown in Table 3. The comparative effectiveness results for ROM are visualized using interval plots in Fig. 4.

### Safety (adverse effects and severe adverse effects)

Of the 42 included studies, 37 with data suitable for adverse effect and severe adverse effect comparison were used for analysis. The SUCRA percentage showed that the SV approach was most likely the best (with a SUCRA value of 80.0 and a mean rank of 2.4) and the MSV approach was most likely the worst (with a SUCRA value of 20.0 and a mean rank of 7.3) in terms of adverse effects. Likewise, the SUCRA percentage showed that the Qsplitt approach was most likely the best (with a SUCRA value of 70.0 and a mean rank of 3.1) and the MQS approach was most likely the worst (with a SUCRA value of 20.0 and a mean rank of 7.4) in terms of severe adverse effects. The SUCRA values and mean rank are summarized in Table 2. A graphical representation of the SUCRA values is provided in Fig. 3. The comparative effectiveness results for adverse effects and severe adverse effects are shown in Table 4. The comparative effectiveness results for adverse effects and severe adverse effects are visualized using interval plots in Fig. 4. There was no evidence of inconsistency between direct and indirect estimates (adverse effects, *P* =.84; severe adverse effects, *P* =.99).

**Table 4:** Network meta-analyses comparison between results of adverse events. Data were pooled odds ratio (OR) and its related 95% CI. Adverse effect: deep vein thrombosis, nerve palsy, superficial hematoma, wound infection, skin bulla; Severe adverse effect: fracture, bleeding (joint hematoma, hemarthrosis), reoperation (revision, manipulation, arthroscopic release), joint infection

Comparison	Adverse effect	Severe adverse effect
MQS vs. MMV	0.51 (0.13, 2.10)	1.12 (0.24, 5.04)
MMP vs. MMV	0.87 (0.54, 1.62)	0.80 (0.31, 1.75)
MSV vs. MMV	2.93 (0.24, 39.15)	2.71 (0.19, 37.18)
MV vs. MMV	2.18 (0.47, 10.32)	1.03 (0.12, 8.39)
QS vs. MMV	1.08 (0.37, 3.27)	1.20 (0.41, 3.43)
MP vs. MMV	1.31 (0.29, 5.73)	1.14 (0.13, 7.84)
SV vs. MMV	1.10 (0.04, 55.21)	0.31 (0.06, 8.84)
Qsplitt vs. MMV	0.49 (0.21, 1.26)	0.64 (0.25, 1.44)
MMP vs. MQS	1.66 (0.46, 6.82)	0.74 (0.13, 3.33)
MSV vs. MQS	5.49 (0.34, 102.54)	2.43 (0.10, 48.18)
MV vs. MQS	4.26 (0.69, 24.20)	0.88 (0.13, 8.71)
QS vs. MQS	2.00 (0.37, 11.76)	1.08 (0.20, 6.25)
MP vs. MQS	2.63 (0.49, 12.70)	1.02 (0.19, 7.30)
SV vs. MQS	2.06 (0.06, 133.11)	0.35 (0.02, 11.29)
Qsplitt vs. MQS	0.95 (0.22, 5.12)	0.58 (0.05, 3.16)
MSV vs. MMP	3.30 (0.29, 44.17)	3.60 (0.28, 48.11)
MV vs. MMP	2.47 (0.46, 12.70)	1.33 (0.12, 12.10)
QS vs. MMP	1.14 (0.37, 4.03)	1.58 (0.68, 3.92)
MP vs. MMP	1.50 (0.34, 6.96)	1.40 (0.17, 11.15)
SV vs. MMP	1.18 (0.03, 65.19)	0.51 (0.04, 12.75)
Qsplitt vs. MMP	0.54 (0.14, 5.14)	0.78 (0.27, 2.60)
MV vs. MSV	0.81 (0.02, 15.71)	0.34 (0.05, 10.67)
QS vs. MSV	0.35 (0.01, 6.11)	0.50 (0.07, 7.00)
MP vs. MSV	0.46 (0.02, 9.07)	0.39 (0.01, 10.49)
SV vs. MSV	0.38 (0.01, 41.74)	0.14 (0.01, 8.32)
Qsplitt vs. MSV	0.21 (0.05, 2.82)	0.24 (0.01, 3.48)
QS vs. MV	0.52 (0.09, 3.21)	1.20 (0.14, 12.14)
MP vs. MV	0.60 (0.20, 2.25)	1.04 (0.07, 11.99)
SV vs. MV	0.52 (0.01, 33.35)	0.33 (0.02, 16.16)
Qsplitt vs. MV	0.18 (0.02, 1.40)	0.56 (0.05, 5.75)
MP vs. QS	1.25 (0.25, 7.86)	0.87 (0.15, 7.80)
SV vs. QS	0.98 (0.07, 61.41)	0.26 (0.03, 8.43)
Qsplitt vs. QS	0.52 (0.07, 2.11)	0.53 (0.13, 1.87)
SV vs. MP	0.84 (0.02, 52.70)	0.35 (0.01, 13.83)
Qsplitt vs. MP	0.38 (0.11, 2.07)	0.59 (0.08, 4.58)
Qsplitt vs. SV	0.42 (0.00, 26.96)	1.64 (0.03, 47.59)

*MP* Medial parapatellar, *MMP* Minimally invasive medial parapatellar, *MV* Midvastus, *MMV* Minimally invasive midvastus, *QS* Quadriceps sparing, *MQS* Minimally invasive quadriceps-sparing, *SV* Subvastus, *MSV* Minimally invasive subvastus, *Qsplitt* Quadriceps splitting, *CI* Confidence interval

## Discussion

The main findings of this NMA were that the MMV and MQS which was minimally invasive TKA approaches led to better outcomes in terms of pain score and ROM. However, the conventional SV and Qsplitt approaches resulted in better outcomes in terms of safety.

The reduction of postoperative pain after TKA was important because it could inhibit early ambulation and ROM [67–69]. According to this NMA, the MMV approach was more effective in pain relief than others. These findings may be attributable to the minimization of soft tissue dissection. Minimally invasive TKA avoids extensive quadriceps disruption, patellar eversion, and dislocation of the tibiofemoral joint [33, 70]. Furthermore, in the MMV approach, the vastus medialis muscle is divided with its muscle fiber as during the MV approach but the division length does not exceed 2 cm [9, 71]. Additionally, the MMV approach might produce better visualization of the knee joint than other minimally invasive approaches by minimally dividing the vastus medialis muscle for lower chance of injury of adjacent soft tissue and correct implant positioning [36, 72]. These factors could lead to less damage to soft tissue so that the pain relief effect will be significant. However, the SUCRA percentage revealed that the MMP approach led to the worst pain relief. These results could be explained by insufficient protection of the patellar vascularity using the MMP method. Although the MMP approach limits the length of arthrotomy, there remains the possibility of patella blood supply disruption due to an incision into the quadriceps tendon, whereas other approaches carry a lower chance of such disruption because they were developed to avoid damage to the patella [1, 9, 73, 74]. When combining these factors, the MMP approach carries no benefits for reduction of pain.

The recovery of the ROM and function of the knee joint after TKA is an important factor for patient satisfaction [75–77]. According to this NMA, the MP approach, which is commonly used for TKA, is most likely the worst approach in terms of ROM. This finding is supported by the possible damage to the quadriceps tendon to lead to eversion of the patella and negative effect on the functional outcome of TKA with MP [3, 78, 79]. To preserve the extensor mechanism, the QS approach was developed by Tria and Coon [13]. Though QS and SV approaches are similar in terms of avoiding violation of the quadriceps tendon, the QS approach does not allow an incision below the vastus medialis oblique muscles, while the SV approach is continued along the lower border of the vastus medialis oblique muscles [10, 14]. Furthermore, the QS approach does not require patellar eversion, which can adversely affect the ROM of the knee joint after TKA [79]. For these reasons, the results of this NMA showed that MQS is most likely the best approach for ROM.

Although minimally invasive TKA had advantages of pain relief and ROM recovery, the SUCRA percentage showed that minimally invasive TKA led to worse outcomes in terms of safety. A proposed explanation of these results is the poorer visual field of a minimally invasive technique than a conventional technique [15, 16]. Because of the limited visual field, the surgery time of minimally invasive TKA is generally longer than that of conventional approaches [80]. Furthermore, stronger tension on the wound edge from retraction could occur through a movable window due to the restricted visual field [15, 81]. For these reasons, minimally invasive TKA could lead to complications such as deep vein thrombosis, wound infection, or delayed wound healing [62, 81]. Another consternation was malpositioning of the implant. Although specially designed instruments are often used to increase limited vision, bone resection and prosthesis positioning are more technically demanding and could lead malpositioning of the implant and failure of primary TKA [14, 82, 83]. While minimally invasive TKA showed better outcomes in pain and ROM, their lower SUCRA values for safety may reflect the technical difficulty and learning curve associated with these techniques. Previous studies have reported higher complication rates during early adoption [84, 85]. This aspect, often overlooked in prior NMAs, warrants further investigation.

This NMA had several limitations. First, we would have missed studies that were not indexed in our selected databases. Second, both the use of an implant for TKA and the surgical procedure chosen varied depending on the surgeon. Postoperative pain management and rehabilitation protocols were also different among trials, which might affect the results. Third, the details of each patient’s medical history, such as preoperative varus or valgus alignment, underlying diseases, obesity, or medication information, were not analyzed because of insufficient data, and these factors may have considerable influence in TKA.

Although several previous network meta-analyses have investigated surgical approaches to TKA, our study extends prior work by including a broader set of nine approaches and analyzing not only functional outcomes such as pain score and ROM, but also adverse effects [70, 86].

## Conclusion

According to this NMA, minimally invasive approaches of TKA led to better outcomes in terms of pain score and ROM, while conventional approaches led to better outcomes in terms of safety. Therefore, orthopedic surgeons should consider various factors when choosing the TKA approach.

## Data Availability

The datasets used and/or analysed during the current study are available from the corresponding author on reasonable request.

## Abbreviations

MP: Medial parapatellar
MV: Midvastus
SV: Subvastus
MMV: Minimally invasive midvastus
QS: Quadriceps sparing
NMA: Network meta-analysis
ROM: Range of motion
TKA: Total knee arthroplasty
MMP: Minimally invasive medial parapatellar
MSV: Minimally invasive subvastus
MQS: Minimally invasive quadriceps-sparing
Qsplitt: Quadriceps splitting
CI: Confidence interval
KSS: Knee society score
HSS: Hospital for special surgery score
SUCRA: Surface under the cumulative ranking curve
